# Factors Influencing Medical Students’ Specialty Choice: Insights to Address the Evolving Primary Care Gap

**DOI:** 10.7759/cureus.88748

**Published:** 2025-07-25

**Authors:** Ana Angeli, Jonathan Ryan, Christian Palacios, Ghaith Al-Eyd

**Affiliations:** 1 Department of Medical Education, Nova Southeastern University's Dr. Kiran C. Patel College of Allopathic Medicine, Fort Lauderdale, USA

**Keywords:** medical specialty, physician shortage, prestige, primary care medicine, professional identity formation

## Abstract

Introduction

The current and projected nationwide shortage of primary care physicians necessitates studying factors that could influence students’ specialty choice for their future medical practice. This study evaluates the influence of potential factors involved in students’ decision-making process regarding specialty selection and proposes appropriate mitigations to enhance interest in primary care specialties.

Methods

Students completed an anonymous 43-question online survey distributed across three medical schools in Florida and California. The survey was sent to all medical students at these institutions, regardless of academic year. It included questions on demographics, specialty interests, perceived external prestige, financial goals, clinical experiences, and personal hobbies. Influential factors were measured using Likert scales. To identify students interested in primary care, we included those who ranked Family Medicine, Pediatrics, or Internal Medicine among their top three specialty choices. The survey was developed through an iterative process among the authors, with a focus on factors such as community perception, peer influence, financial incentives, and clinical exposure.

Results

One hundred students completed the survey, with only 26% (n=26) ranking surgery as their top choice, and 64% (n=64) of students including primary care in their top three choices. Students rated their top choice as having a better reputation in the healthcare field compared to primary care (p = 0.0028). Students also rated their top specialty choices as requiring a higher skill level compared to primary care (p = 0.0015). The median minimum level of income students would be satisfied with as an attending was $250-300k. Students who chose a minimum level of income below $300k were more likely to agree with the statement that a clinical experience made them choose their specialty (p = 0.016).

Conclusion

We found that students’ specialty choices were influenced by income considerations, perceived biases against primary care within the healthcare community, and their own perceptions of their skill levels. Introducing early clinical exposure to primary care, such as incorporating primary care physicians as faculty and providing early hands-on experiences, may help improve perceptions of primary care specialties and foster greater student interest in these fields.

## Introduction

This study aims to examine the influence of income expectations, clinical experiences, perceptions of prestige, and personal interests on medical students’ specialty choices, with a focus on primary care. Statistical trends have provided increasing evidence that the current physician shortage is expanding. A model created by Zhang X et al. (2020) predicts that there will be a shortage of 139,160 physicians in the US by the year 2030 [1]. This model uses different grades to compare the national mean shortage ratio (physician shortage per 100,000 people) to that of different states. Grade “C” is given to the national mean, and the grades of “B” and “D” are defined as being ±1 SD, while “A” and “F” are defined as being ±2 SDs. Using this scale, 23 states will have a physician shortage ratio of “D” or “F” by 2030, compared to 4 states in 2017. The states with the greatest estimated physician shortages are California, Florida, and Texas, in that order. Florida is also one of the fastest-growing states, its population is expected to increase by 30% in the next 10 years.

The increasing population includes a rapidly growing proportion of older adults who naturally require frequent use of the healthcare system, which necessitates a parallel growth in the physician workforce. However, according to the Florida Health Department’s 2022 Physician Workforce Annual Report, 9.7% of physicians are planning to retire in the next 5 years, and the two specialties with the greatest number of retiring physicians are internal medicine and family medicine [2]. In addition, 4.7% of physicians are planning to relocate to another state, with the highest proportion coming from internal medicine and family medicine. This means that primary care specialties, which are already in high demand, will become increasingly overburdened. Only 2% of physicians work in Florida’s 30 rural counties, despite these areas being home to 9% of the population [3]. Considering these areas rely heavily on primary care physicians, as they often lack access to specialists and large tertiary care centers, this shortage is particularly concerning. The Physician Workforce Advisory Council has advised the Florida Health Department to implement strategies to retain graduates in internal medicine and family medicine because of the specific needs of the population [2].

The plan to address the expected gap in primary care physicians should start as early as when medical students begin shaping their future specialty choices. Pfarrwaller E et al. (2017) designed an expanded conceptual framework of medical students’ primary care career choice [4]. This framework has informed us on which influential factors to more closely examine in our study. According to this model, students’ preexisting characteristics place them into four categories based on initial interest: primary care committed, primary care positive, undecided, and non-primary care committed. During their time in medical school, students consider self-efficacy and expected outcomes in each field. This means that students judge their own ability to complete tasks in an assigned field, as well as the rewards (financial, social, or emotional) of a given specialty. Interest in a specialty grows into the formation of career goals, which lead to related actions (i.e., participation in electives or academic organizations) that affect academic performance and further inform judgments about self-efficacy and expected outcomes [4]. According to Babbott D et al. (1991), most medical students choose a specialty in their third or fourth years of undergraduate medical education [5,6]. However, students may drop out or change their specialty during their training, which can be a source of distress and missed career opportunities [7].

According to Pfarrwaller E et al. (2017), there are several external factors that can hierarchically affect the process of students’ decision-making related to their healthcare career choices. In order of importance, these factors are the microsystem, mesosystem, exosystem, and macrosystem [4]. The microsystem represents factors that directly impact the student, such as interactions with professors, role models, family, and engagement with the curriculum. The mesosystem represents the interplay between each part of the microsystem, such as how family life affects schoolwork. The exosystem represents indirect influences on the student, such as physician shortages and job opportunities. The macrosystem represents societal values that influence students, such as medical school culture and healthcare policies. It is apparent that many of these external factors are related to the learning environment, including the faculty and curriculum. Hence, medical schools have a unique opportunity to implement interventions to encourage participation in fields with physician shortages, such as primary care.

Given this estimated nationwide shortage of primary care doctors, especially in the state of Florida, where our institution is located, we conducted a multi-institution study to identify factors that influence students’ medical specialty choices. Our study aims to address this gap by identifying factors that influence medical students’ specialty choice and suggesting potential interventions to increase interest in primary care. We hypothesize that desired income, as well as clinical experience, will play a significant role in determining interest in primary care.

The results reported in this article were previously presented as a meeting abstract at the 2023 Annual RISE Conference, Dr. Kiran C. Patel College of Allopathic Medicine, Nova Southeastern University, on September 8, 2023. The abstract was also orally presented at the “Professional Identity Development and Becoming a Physician” session at the 2024 AAMC Learn Serve Lead Meeting on November 11, 2024.

## Materials and methods

An anonymous online survey consisting of 43 items was distributed across three institutions from July 2023 to November 2024: Nova Southeastern University (NSU), Dr. Kiran C. Patel College of Allopathic Medicine (Fort Lauderdale, Florida); University of Central Florida, College of Medicine (Orlando, Florida); and California University of Science and Medicine, School of Medicine (Colton, California). Participants included first-, second-, third-, and fourth-year medical students, as well as pre-medical students. The states of Florida and California are among those projected to have the highest levels of physician shortages across the United States in the next 10 years [1]. Invitations were sent to multiple universities located in these two states, and the questionnaire was distributed to students enrolled in the medical schools of the universities that accepted the invitation. We did not limit our survey to a particular academic year in order to broadly capture students’ opinions across their time in medical school. We felt this broad inclusion likely enhances the generalizability of our findings. The survey was developed through an iterative process among the authors, with a focus on factors such as community perception, peer influence, financial incentives, and clinical exposure. Questionnaires were delivered via student email by their own institutions without additional reminders, which may have limited our response rate. This study was reviewed and granted exempt status by the NSU Institutional Review Board.

Based on the purpose of the study, the questionnaire was uniquely designed by the research team to collect relevant information on the demographics of the study sample, specialty interests and disinterests, the confidence level of specialty choices, as well as potential external factors that could influence those choices. The survey can be found in Appendix 1. Guided by the Association of American Medical Colleges Careers in Medicine Specialty Profiles resource, the survey included specialty options of 27 accredited surgical and non-surgical specialties [8]. Survey responses on specialty choice were classified as: A) Students pursuing a surgical or non-surgical specialty based on their top-ranked specialty choice. This is because such broad categories would not provide useful information when combined with all three ranked specialties. B) Students were considered interested in primary care if they ranked a primary care specialty (Family Medicine, Pediatrics, or Internal Medicine) among their top three specialty choices. Hobbies included in the survey were chosen based on their association with different medical specialties. For example, in our institution, we tend to associate athleticism with surgical specialties and literature with psychiatry. This questionnaire was developed based on a previous study by Clinite KL et al. as well as our own experiences as medical students and educators [9]. It was not previously piloted or validated.

Statistical analyses were conducted using Excel (version 2506) and SPSS (version 29.0.2.0). We performed general descriptive statistics across subgroups, as well as t-tests and ANOVA to assess the significance of selected variables, including interest in surgical specialties, primary care specialties, and minimum desired income, as described below. Students self-reported their race, ethnicity, location of upbringing, relationship status, gender, and year in school. While we did not perform multivariable analyses, we conducted subgroup analyses to assess differences between students interested and not interested in primary care specialties.

To compare students who ranked a surgical specialty as their top choice, we conducted unpaired t-tests on mean Likert scale scores for questions assessing interest in art, reading/writing, cooking/baking, puzzles, video games, and exercise/sports. In order to perform unpaired t-tests, we treated the Likert scale scores as interval data on a scale from 1 to 5. We chose to perform this analysis rather than Mann-Whitney U tests, given our large sample size and the ongoing debate regarding the most appropriate method [10].

Desired income was assessed through the question: “What is the minimum level of income you would be satisfied with as an attending?” with response options provided in $50,000 intervals. To explore how income expectations influenced responses to clinical experience questions, we stratified students into two groups based on whether they desired more or less than $300,000 annually; this threshold was selected arbitrarily, as it approximated the median response in our sample.

To examine perceptions of primary care, we performed unpaired t-tests comparing students who ranked a primary care specialty (Family Medicine, Pediatrics, or Internal Medicine) in their top three choices with those who did not. These comparisons focused on responses related to income expectations, perceived skill level in primary care, and the perceived reputation of primary care within both the general community and the healthcare field.

Lastly, to assess how students from different specialty interests viewed primary care, we grouped respondents based on their top-ranked specialty into the following categories: surgery, family medicine/pediatrics, ancillary specialties (anesthesiology, pathology, radiology), internal medicine, and other (including dermatology, emergency medicine, neurology, physical medicine and rehabilitation (PM&R), and psychiatry). This grouping strategy allowed for meaningful categorization while preserving sufficient statistical power to draw conclusions. A p-value less than 0.05 was considered statistically significant.

## Results

A total of 100 students from three institutions completed the survey. Table 1 shows the general descriptive statistics on students' backgrounds, race, ethnicity, and other personal information. The majority of respondents were Caucasian (59%, n=59), non-Hispanic (85%, n=85), partnered (54%, n=54), female (57%, n=57), and grew up in a suburban area (79%, n=79).

**Table 1:** Demographics of students who completed the survey. Data are presented as N (%).

Factor	# of Students (%)
Total	100
Race	
- Black	7 (7%)
- Caucasian	59 (59%)
- East Asian	9 (9%)
- South Asian	16 (16%)
- Other	9 (9%)
Ethnicity	
- Hispanic	15 (15%)
- Non-Hispanic	85 (85%)
Location of Upbringing	
- Rural	11 (11%)
- Suburban	79 (79%)
- Urban	10 (10%)
Relationship Status	
- Married	9 (9%)
- Partnered	54 (54%)
- Single	37 (37%)
Gender	
- Male	43 (43%)
- Female	57 (57%)
Year of School	
- Premedical	10 (10%)
- First Year	33 (33%)
- Second Year	26 (26%)
- Third Year	15 (15%)
- Fourth Year	16 (16%)

**Figure 1:**
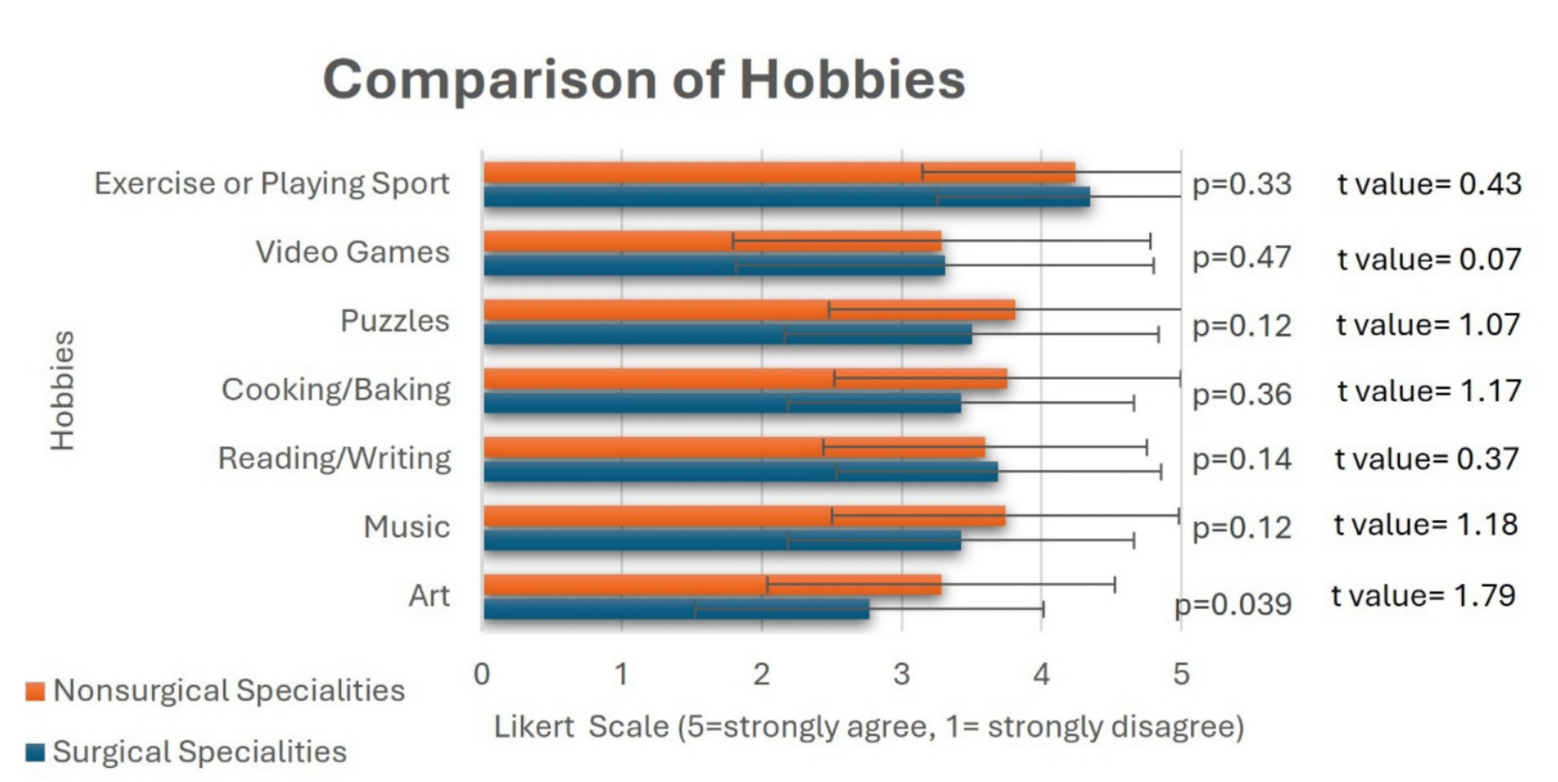
T-tests comparing hobbies between students whose top specialty was surgical vs. non-surgical. The data represent the students’ mean Likert scale scores ± SD for each group (p < 0.05 considered statistically significant). Scale: 1 = strongly disagree, 2 = disagree, 3 = neither agree nor disagree, 4 = agree, 5 = strongly agree.

Only 26% (n=26) of students ranked a surgical specialty as their top choice. Additionally, students interested in surgery reported a higher median desired minimum income (median: $300-$349k) compared to those who were not interested in surgery (median: $250-$299k). For most hobbies, there were no significant differences between students interested in surgery and those who were not (Figure 1). However, students desiring a surgical specialty (mean score: 2.77) were less interested in the arts compared to those not pursuing surgery (mean score: 3.28, p=0.039, Figure 1). Additionally, we found no statistically significant differences in hobby interests between students who ranked a primary care specialty in their top three choices and those who did not.

**Figure 2:**
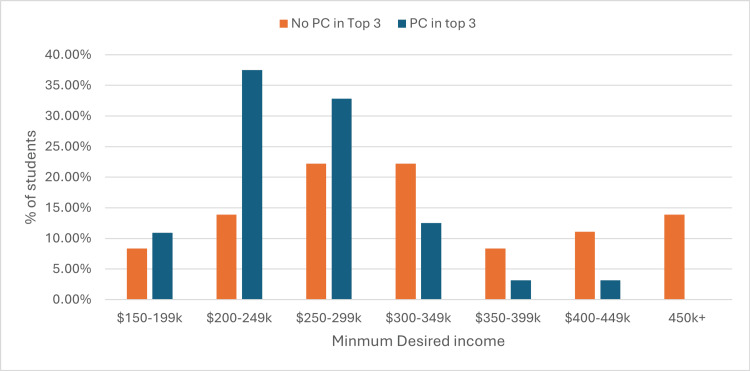
Minimum desired income of students interested in primary care specialties vs. students not interested in primary care specialties. The figure shows the percentage of students within each self-reported income bracket. PC: Primary care.

**Figure 3:**
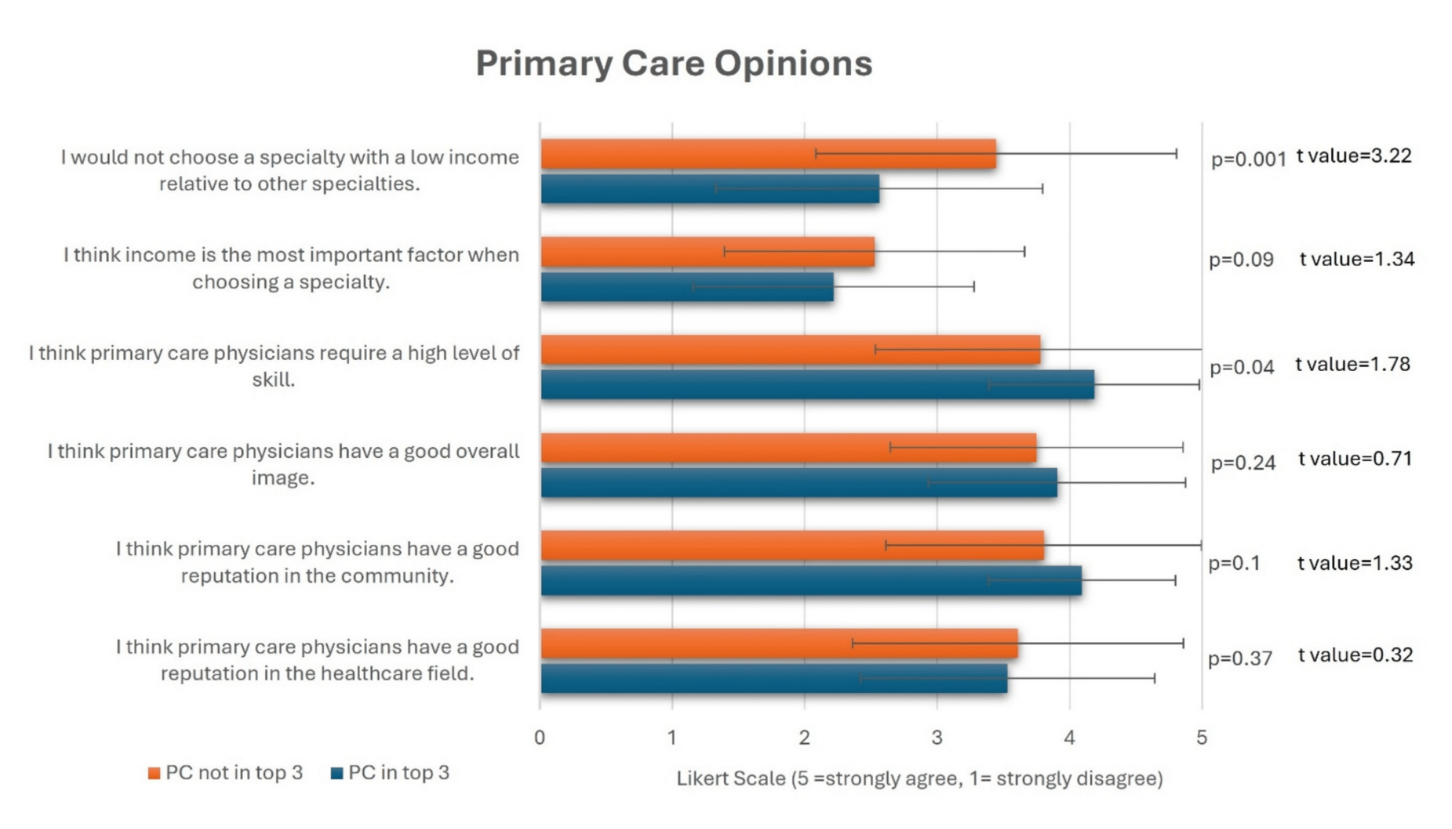
T-tests comparing primary care opinions between students interested in primary care and students not interested in primary care. This figure presents the students' mean Likert scale scores in each group ± SD (p < 0.05 is considered significant). 1 = strongly disagree, 2 = disagree, 3 = neither agree nor disagree, 4 = agree, 5 = strongly agree. PC: Primary care.

Interestingly, 64 students (64%) ranked a primary care specialty among their top three choices, and they had a lower median desired minimum income ($250-$299k) compared to those who did not prioritize primary care ($300-$349k, Figure 2, Mann-Whitney U test, p < 0.001). Students not interested in primary care (mean Likert score: 3.44) were more likely to agree with the statement, “I would not choose a specialty with a low income relative to other specialties,” compared to those interested in primary care (mean Likert score: 2.56, p=0.001). However, both groups expressed high levels of respect for primary care physicians (Figure 3). Students interested in primary care were more likely to agree with the statement, “I think primary care physicians require a high level of skill” (p=0.04).

Comparing students interested in primary care to those who were not, primary care-interested students were more likely to come from suburban backgrounds (81% vs. 75%; n=52 vs. n=27; Χ² p=0.46) and less likely to come from urban backgrounds (8% vs. 14%; n=5 vs. n=5; Χ² p=0.33), with equal representation from rural areas (11% each, n=7 and n=4; Χ² p=0.98). Additionally, students interested in primary care were more likely to be partnered (56% vs. 50%; n=36 vs. n=18; Χ² p=0.55), married (9% vs. 8%; n=6 vs. n=3; Χ² p=0.86), and less likely to be single (34% vs. 42%; n=22 vs. n=15; Χ² p=0.47) compared to those not interested in primary care.

**Figure 4:**
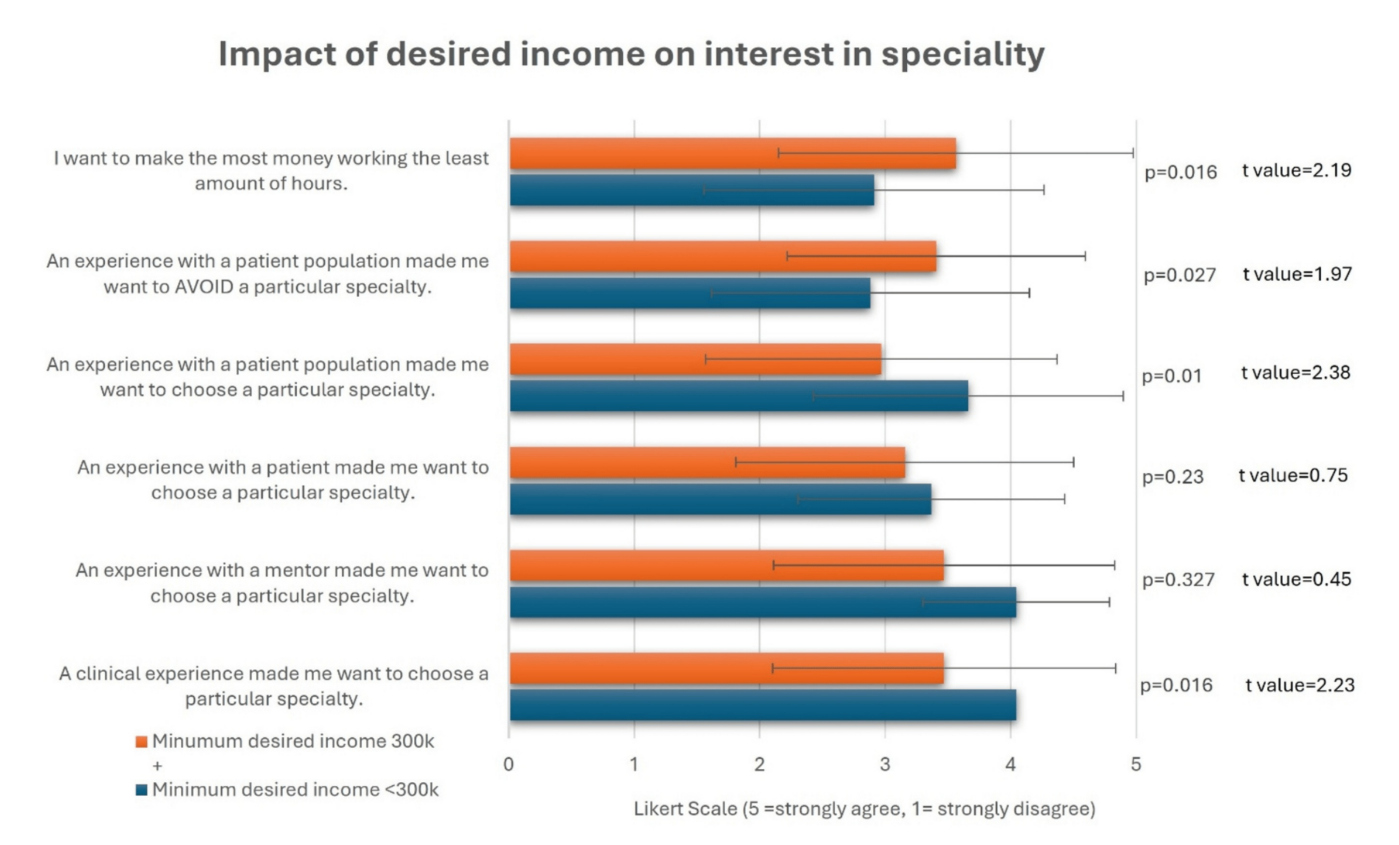
T-tests comparing the influence of clinical experience between students with a minimum desired income <300k and students with a minimum desired income >300k. This figure presents the students' mean Likert scale scores in each group ± SD (p < 0.05 is considered significant). 1 = strongly disagree, 2 = disagree, 3 = neither agree nor disagree, 4 = agree, 5 = strongly agree.

Another analysis showed how students’ minimum desired income influenced their choice of specialty. Overall, 20% (n=20) of students agreed or strongly agreed with the statement, “I think income is the most important factor when choosing a specialty.” A total of 68 students (68%) were comfortable with a minimum salary below $300k, while 32 students (32%) desired a minimum salary of $300k or more. Students who were comfortable with a salary below $300k were more likely to be influenced by clinical experiences and interactions with specific patient populations, as offered by the educational program, when selecting their specialty (Figure 4). This suggests that interventions should ideally be targeted toward students who are satisfied with a more modest income. In contrast, those who desired a salary of $300k or more were more likely to base their choice on avoiding certain specialties due to experiences with patient populations, as well as prioritizing earning the maximum income for their time worked.

**Figure 5:**
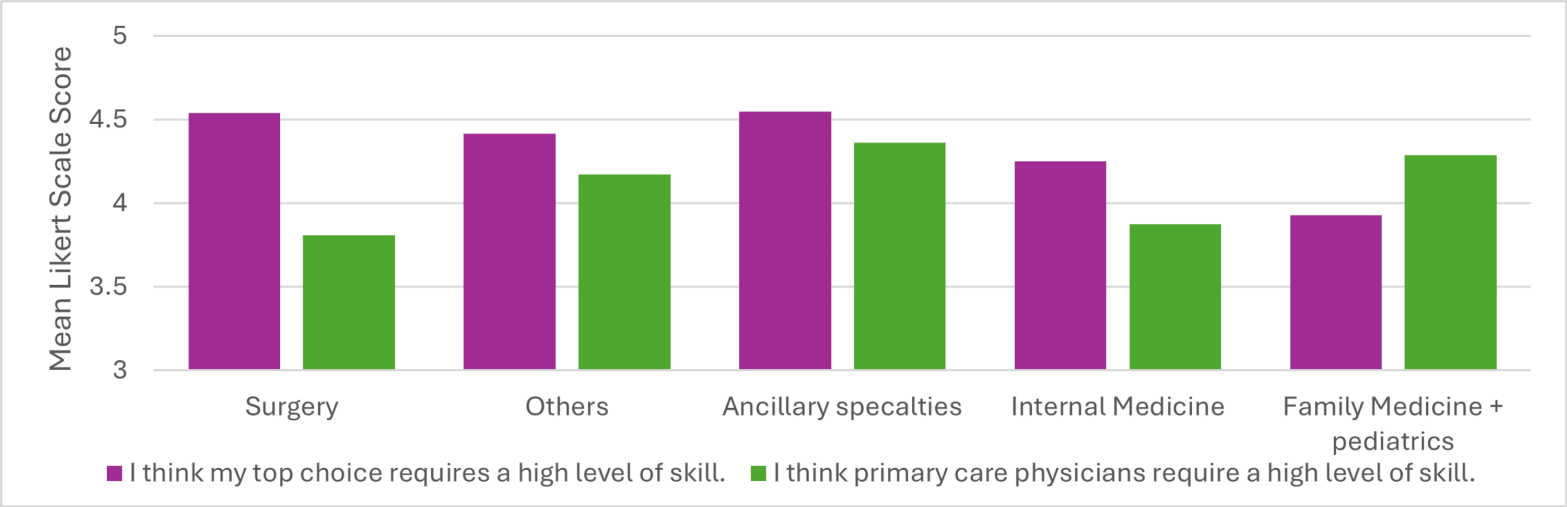
Skill required in primary care specialties compared to students’ top specialty. Data represent the mean Likert scale scores for each specialty based on two separate survey questions. Surgical specialties (n = 26), family medicine and pediatrics (n = 14), ancillary specialties (anesthesiology, pathology, radiology) (n = 15), others (dermatology, emergency medicine, neurology, PM&R, psychiatry) (n = 29), and internal medicine (n = 16). PM&R: Physical Medicine and Rehabilitation. 1=strongly disagree, 2=disagree, 3=neither agree nor disagree, 4=agree, and 5=strongly agree.

**Figure 6:**
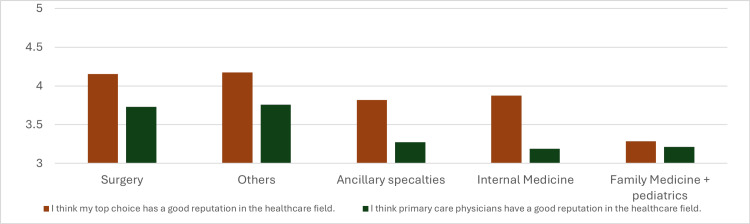
Reputation in the healthcare field of primary care specialties compared to students’ top specialty. Data represent the mean Likert scale scores for each specialty based on two separate survey questions. Surgical specialties (n = 26), family medicine and pediatrics (n = 14), ancillary specialties (anesthesiology, pathology, radiology) (n = 15), others (dermatology, emergency medicine, neurology, PM&R, psychiatry) (n = 29), and internal medicine (n = 16). 1 = strongly disagree, 2 = disagree, 3 = neither agree nor disagree, 4 = agree, 5 = strongly agree. PM&R: Physical Medicine and Rehabilitation.

Among other reported factors influencing individual specialty preferences is the low self-reported image associated with some specialties. Students’ responses to these topics appeared similar. Most students rated their own top-choice specialty as requiring a higher level of skill compared to primary care physicians (Figure 5, matched two-tailed t-test, p=0.0015, mean difference = 0.39, 95% CI = 0.15-0.63), with this difference being most notable in specialties such as General Surgery and Vascular Surgery. This perceived decrease in skill level is a critical aspect of the reduced interest in primary care. Figure 6 shows that most students perceive their prospective specialty as having a better reputation in the healthcare community compared to primary care (matched two-tailed t-test, p=0.0028, mean difference = 0.38, 95% CI = 0.13-0.63). However, there did not appear to be significant differences in students' general opinions of how their specialty is viewed by the broader community compared to primary care.

## Discussion

This study is one of the few multi-institutional surveys examining medical students’ perceptions of primary care and the factors influencing their specialty choices. This strengthens the generalizability of our findings by including diverse perspectives from different regions. Our study highlights a persistent association among medical students between primary care and lower perceived external prestige. Students rated their preferred specialties as more skilled and reputable than primary care (Figures 5-6). Those who desired higher incomes (>$300k) expressed lower interest in primary care, were more financially motivated, and were less influenced by clinical experiences. However, income alone did not account for this trend, only 20% agreed that income was the most important factor when choosing a specialty. This suggests that students who are satisfied with a more modest income should be the primary targets of long-term interventions to increase interest in primary care, such as mentorship and early clinical exposure. In contrast, 73% of students agreed that a clinical experience influenced their specialty choice, suggesting that meaningful clinical exposure may outweigh financial motivations in shaping student preferences.

Hobbies and personality may also contribute to specialty selection. Students choosing non-surgical specialties were more likely to report an interest in visual arts. This may reflect personality traits such as openness and divergent thinking, which are more common in individuals with artistic inclinations [11, 12]. However, most hobbies (e.g., reading, sports, music) were evenly distributed across specialties, indicating a broader similarity among students regardless of their intended field. This suggests that differences in lifestyle and personality may not be as predictive as often assumed. It is also possible that we did not have enough power to detect further differences in hobbies. A future study with a larger sample size may be necessary to provide more insight.

Prestige

This study uniquely explored prestige by measuring students' perceptions of skill level, professional reputation, and community respect, without explicitly using the word “prestige.” This was important, as our study attempted to limit social desirability bias. While students generally perceived their chosen specialty as more skilled than primary care, there were no significant differences in how they thought society or healthcare workers viewed their field compared to primary care. Given that this perception was most notable among students who opted for General Surgery and Vascular Surgery, specialties that involve explicit motor skills (e.g., surgical procedures) could be subjectively perceived as requiring higher skill levels compared to the implicit, wide range of clinical reasoning skillsets needed for the general practice of primary care. It is also possible that the fast results of a surgical intervention, compared to medical management, contribute to this perception. 

Few studies have examined prestige in this way. Future research could build on this concept by using larger sample sizes, including more pre-medical students, and even including residents from various medical specialties. Clinite KL et al. found that medical students who placed less value on prestige were more likely to be interested in primary care, though prestige was not the most influential factor overall [9]. In a longitudinal study, Ladha FA et al. found that fourth-year students were more likely to express interest in primary care than first-years, suggesting that interest in primary care can grow over time [13]. They also found that prestige and salary mattered more to those choosing surgical specialties, while non-surgical students prioritized family, location, and match confidence.

International data reinforce these findings. In Australia, students ranked surgery as the most prestigious field, while public health and non-specialist roles ranked lowest [14]. In Canada, Wright B et al. found that career choice was largely influenced by five factors: lifestyle, societal orientation, prestige, hospital orientation, and scope of practice. Family medicine students emphasized lifestyle and societal contribution over prestige [15]. Our study supports the notion that perceived prestige is often shaped by a combination of community opinion, the views of fellow healthcare professionals, and an individual’s internal perception of skill. Despite these subjective factors, they continue to play a significant role in influencing the decision to pursue, or avoid, a career in primary care. Considering we found no differences in how primary care was perceived by the community compared to other specialties, it is possible that people outside of the healthcare field are more appreciative of the work that primary care physicians do. This underscores the importance of interventions implemented by medical schools to alleviate negative biases toward primary care.

Financial goals

In our study, while financial goals were associated with decreased interest in primary care, most students selected a desired minimum income below $300k. As in Clinite KL et al., students who prioritized income were less likely to consider primary care, although the top-rated career factor was enjoyment of the work itself [9]. Financial preferences may reflect both perceived workload and lifestyle, as students associate higher-paying specialties with greater effort and responsibility. Based on our findings, these perceptions are likely formed early in a student’s career, during pre-medical shadowing and the pre-clerkship years of medical school. This further emphasizes the need for early clinical exposure to primary care in a variety of settings.

Clinical experience

This study showed that when income was not the main factor affecting specialty choice, clinical experiences and interactions with specific patient populations offered by the educational program were influential in students’ decisions. Across studies, positive experiences in a specialty, through rotations, mentorship, or role modeling, strongly shaped student interest. Du J et al. found that all surgical respondents had positive prior experiences during surgical rotations, compared to 71% of those who ultimately chose non-surgical paths [16]. Hin HK et al. (2007) surveyed 118 medical students, residents, and physicians in British Columbia and found that “personal interests” and “previous positive clerkship experience” were the most influential factors in choosing family medicine [17]. Respondents who had previously had a mentorship experience rated this as a more influential factor than those without. Additionally, physicians demonstrated a greater appreciation for mentorship than students, possibly indicating that these experiences become more valuable later in a physician’s career. It is important to note that we cannot interpret causality from these findings, and it is possible that students self-select for experiences that reinforce preexisting preferences.

Stagg P et al. (2012) conducted a systematic review of 36 articles and found that preceptors who are rated as high-quality teachers can influence students’ career choices in as little as three weeks [18]. This influence can increase student interest in a field by up to four times. Conversely, preceptors who were rated as negative role models, poor teachers, or lacking discipline-specific knowledge decreased student interest in that specialty. Increased duration of preceptorship also increased student interest, especially in primary care specialties. Most schools use student surveys to monitor the quality of preceptorships, but we believe this method is insufficient, as many students may not feel comfortable reporting negative experiences. They may also be unaware of what to expect from a high-quality clinical experience. Many students have difficulty distinguishing whether their negative clinical experience was due to the site or the specialty itself. Medical schools may consider intermittent, unannounced visits by administration to ensure that preceptors are meeting institutional quality standards. This may be costly and time-consuming, and preceptors may be resistant to this level of observation. However, we believe this is the most effective way for medical schools to monitor education at clinical sites. 

Mentorship and longitudinal programs also significantly increase primary care match rates. Indyk D et al. (2011) designed a four-year mentorship program that increased student matching into primary care specialties by over one-third [19]. Students with an initial interest in primary care were assigned to a primary care clinician and met with them monthly during pre-clerkship years and quarterly during clerkships. They also participated in monthly didactic conferences, engaged in community-based primary care research projects, and attended national primary care conferences. Compared to students with an initial interest in primary care who were not assigned a mentor, those who were matched into primary care specialties at greater rates.

For example, Indyk D et al. reported that first-year students paired with a primary care mentor throughout medical school matched into primary care specialties at significantly higher rates [18]. Phillips J et al. noted that while senior students had more negative perceptions of primary care work-life, they were paradoxically more likely to pursue it than juniors, perhaps due to positive role models or deeper clinical exposure [20]. This suggests that students gain more realistic expectations and potentially a deeper appreciation of primary care as they gain more experience. Thus, given our findings that mentors play a profound role in influencing primary care choices, it could benefit medical schools to institute early mentorship programs with primary care physicians. Pfarrwaller E et al.'s review supports the idea that longitudinal, multi-component programs are the most effective in increasing student interest in primary care [21]. These programs involved preclinical preceptorships, family medicine faculty advisors, clinical rotations at rural or regional locations, family medicine workshops, and community service projects. In support of our findings on the significance of clinical experience, these results suggest there should be more targeted early exposure preceptorships and clinical rotations in primary care medicine. High-quality primary care preceptorships during pre-clerkship years, and even access to a student-run primary care clinic, may be key to increasing interest in these specialties.

Hobbies and stereotypes

Although medicine is rife with stereotypes, little research exists on the role of hobbies in specialty choice. This is an important area of study, as stereotypes may lead to self-selection or self-exclusion, which can play a prominent role in specialty choice. Our study found limited significant associations between students’ hobbies and their interest in surgical specialties. This finding was surprising but understandable, considering our modest sample size and the variety of surveyed specialties. Notably, students who liked surgery were less likely to be interested in the arts. Shay T et al. found that plastic surgeons were more likely to engage in creative hobbies such as music and crafts [22]. However, Schnapp BH et al. found that even in Emergency Medicine (EM), where stereotypes suggest applicants are outdoorsy or athletic, the most common hobbies were similar to those of applicants in psychiatry and plastic surgery [23].

Harendza S and Pyra M found that stereotypes about internists as cautious and analytical were reinforced over time, likely shaped by the educational environment [24]. Shappell E and Schnapp B caution against the overuse of the term “fit” in residency selection, which may mask bias and discourage diversity [25]. “Fit” often refers to shared identity markers, such as personality or background, that can influence both program decisions and student perceptions of where they belong. This may unintentionally reinforce exclusionary norms in specialty culture. Program directors may redefine “fit” as having a shared mission in order to make their programs more inclusive. Ben-Ner A et al. found that identity plays a key role in forming workplace in-group preferences [26]. Kinship, politics, religion, and personal interests may influence both who gets selected and how students envision themselves fitting into a particular specialty.

Our study investigated hobbies as a factor that may relate to the concept of “fit” influencing specialty choice. If there were stark differences in hobbies between specialties, perhaps they could be used to guide students who are unsure of their path. However, our research suggests that hobbies, and the concept of “fit”, may not play a large role in specialty choice. Thus, we should encourage students to pursue whichever specialty interests them, regardless of whether they feel they fit a certain stereotype. This message should be reinforced by medical school faculty and academic advisors, who should actively challenge stereotypes.

Putting it all together

This study suggests that while financial considerations and prestige matter, clinical experience and cultural perceptions of belonging play a larger role in specialty selection. Medical schools can address this in three key ways: 1) Preceptorships during pre-clerkship, 2) Primary care mentors for interested students, and 3) Bias training for students and faculty. Interventions aimed at increasing interest in primary care should focus on raising students’ awareness about the vital role of primary care within the healthcare spectrum through early, longitudinal clinical exposure with high-quality mentors and the promotion of positive role models. In addition, addressing subtle biases and reinforcing the skill and value associated with primary care could reshape the culture surrounding these specialties. This could be achieved through curricular reforms that integrate relevant learning experiences, such as community service projects and real-life case study exercises. In-person observation of clinical preceptors by medical school administration may also be necessary to ensure that these values and quality standards are maintained. This is not meant to take away autonomy from preceptors but to build a more structured clerkship experience that reflects the values of the institution.

Limitations and strengths

Our study is limited by its modest sample size and its inclusion of only three medical schools, which may limit generalizability. Distribution was restricted to internal channels to ensure validity but may have introduced selection bias. Surveys were distributed locally by each participating institution via email; therefore, we could not determine how many students viewed the survey. Internal distribution increases validity by ensuring the authenticity of respondents and having institutional oversight, as opposed to posting the survey on a public online student platform. The cross-sectional nature of the study also prevents the assessment of changes in perception over time. Future studies may address this limitation by following students throughout their medical education, and potentially even into graduate medical education. Additionally, our survey was not previously piloted or validated. Future research could refine and validate the survey instrument. Lastly, we did not apply statistical corrections such as a Bonferroni adjustment due to our limited sample size; however, this may increase the risk of false positives. Nevertheless, this study offers several strengths. It includes a detailed analysis of prestige without relying on direct labeling, which greatly reduces social desirability bias. This is a key strength that should be replicated in future studies. It also incorporates lesser-studied variables like hobbies and surveys students from both public and private institutions in diverse geographic regions. These design elements provide a nuanced understanding of how students view primary care and what interventions may support greater interest in it.

## Conclusions

In this multi-institutional study, we found that students associate a lower perceived external prestige with primary care specialties. This may partially explain the current and predicted shortage of primary care physicians. Addressing perceptions of lower prestige may be key to increasing interest in primary care. While financial goals are important, the lack of interest in primary care cannot be explained by this factor alone. Students may be more strongly influenced by modifiable factors such as clinical experiences than by financial incentives, suggesting that early exposure to primary care may help alleviate the shortage in this field. To mitigate these negative biases, medical schools could implement interventions such as early clinical exposure to primary care, hiring primary care physicians as faculty, and providing bias training related to the perception of different medical specialties. The effectiveness of these interventions may be evaluated through periodic student surveys and by tracking the institution's primary care match rates over time. Early clinical exposure should include preclinical preceptorships, clinical rotations in rural or regional locations, and community service projects. Bias training can be delivered through lectures and case-based exercises and should include students, faculty, and clinical preceptors. Both explicit and implicit bias should be addressed in these interventions. Intermittent observation of preceptors is recommended to ensure that students are receiving high-quality teaching and that bias is not being introduced at clinical sites. Constructive feedback should ideally be delivered in person by administration, fostering a collaborative conversation. The goal of this observation and feedback is to ensure that students receive the educational structure they need to develop their skills and that they are placed in a positive learning environment aligned with the institution’s values.

## Appendices

Appendix 1 

**Table 2:** Survey questions. This questionnaire was specifically developed by the research team for the purposes of this study.

Question Number	Question / Sub-Item	Answer Choice
1	Do you consent to participate in this study?	Yes
		No
2	Where are you in your medical education?	I am a pre-medical student and/or applying to medical schools
		I am a first-year medical student
		I am a second-year medical student
		I am a third-year medical student
		I am a fourth-year medical student
3	Which gender do you identify as?	Female
		Male
		Non-binary
		Other
4	Which race do you identify as?	Caucasian
		Black
		East Asian
		South Asian
		Native American/Pacific Islander
		Other
5	Which ethnicity do you identify as?	Hispanic/Latino
		Non-Hispanic/Latino
6	What type of setting did you live in for the majority of your life up to age 18?	Rural
		Suburban
		Urban
7	Which best describes your relationship status?	Single
		Partner
		Married
8	Please select the specialty you are most interested in.	Allergy and Immunology
		Anesthesiology
		Colon and Rectal Surgery
		Dermatology
		Diagnostic Radiology
		Emergency Medicine
		Family Medicine
		General Surgery
		Hospice and Palliative Medicine
		Thoracic/Cardiac Surgery
		Vascular Surgery
		Internal Medicine
		Medical Genetics and Genomics
		Musculoskeletal Oncology
		Neurological Surgery
		Neurology
		Obstetrics and Gynecology
		Ophthalmology
		Orthopaedic Surgery
		Otolaryngology-Head and Neck Surgery
		Pathology
		Pediatrics
		Physical Medicine and Rehabilitation
		Plastic Surgery
		Public Health and Preventative Medicine
		Psychiatry
		Urology
9	How certain are you of your choice in the previous question?	Not at all certain
		Somewhat certain
		Fairly certain
		Very certain
		Extremely certain
10	Please select the specialty you are second most interested in.	Allergy and Immunology
		Anesthesiology
		Colon and Rectal Surgery
		Dermatology
		Diagnostic Radiology
		Emergency Medicine
		Family Medicine
		General Surgery
		Hospice and Palliative Medicine
		Thoracic/Cardiac Surgery
		Vascular Surgery
		Internal Medicine
		Medical Genetics and Genomics
		Musculoskeletal Oncology
		Neurological Surgery
		Neurology
		Obstetrics and Gynecology
		Ophthalmology
		Orthopaedic Surgery
		Otolaryngology-Head and Neck Surgery
		Pathology
		Pediatrics
		Physical Medicine and Rehabilitation
		Plastic Surgery
		Public Health and Preventative Medicine
		Psychiatry
		Urology
11	How certain are you of your choice in the previous question?	Not at all certain
		Somewhat certain
		Fairly certain
		Very certain
		Extremely certain
12	Please select the specialty you are third most interested in.	Allergy and Immunology
		Anesthesiology
		Colon and Rectal Surgery
		Dermatology
		Diagnostic Radiology
		Emergency Medicine
		Family Medicine
		General Surgery
		Hospice and Palliative Medicine
		Thoracic/Cardiac Surgery
		Vascular Surgery
		Internal Medicine
		Medical Genetics and Genomics
		Musculoskeletal Oncology
		Neurological Surgery
		Neurology
		Obstetrics and Gynecology
		Ophthalmology
		Orthopaedic Surgery
		Otolaryngology-Head and Neck Surgery
		Pathology
		Pediatrics
		Physical Medicine and Rehabilitation
		Plastic Surgery
		Public Health and Preventative Medicine
		Psychiatry
		Urology
13	How certain are you of your choice in the previous question?	Not at all certain
		Somewhat certain
		Fairly certain
		Very certain
		Extremely certain
14	Please select the 3 specialties you are LEAST interested in.	Allergy and Immunology
		Anesthesiology
		Colon and Rectal Surgery
		Dermatology
		Diagnostic Radiology
		Emergency Medicine
		Family Medicine
		General Surgery
		Hospice and Palliative Medicine
		Thoracic/Cardiac Surgery
		Vascular Surgery
		Internal Medicine
		Medical Genetics and Genomics
		Musculoskeletal Oncology
		Neurological Surgery
		Neurology
		Obstetrics and Gynecology
		Ophthalmology
		Orthopaedic Surgery
		Otolaryngology-Head and Neck Surgery
		Pathology
		Pediatrics
		Physical Medicine and Rehabilitation
		Plastic Surgery
		Public Health and Preventative Medicine
		Psychiatry
		Urology
15	How certain are you of your choices in the previous question?	Not at all certain
		Somewhat certain
		Fairly certain
		Very certain
		Extremely certain
16a	I think my top choice has a good reputation in the community.	Strongly disagree
		Disagree
		Neither agree nor disagree
		Agree
		Strongly agree
16b	I think my top choice has a good reputation in the healthcare field.	Strongly disagree
		Disagree
		Neither agree nor disagree
		Agree
		Strongly agree
16c	I think my top choice is actively involved in serving the community.	Strongly disagree
		Disagree
		Neither agree nor disagree
		Agree
		Strongly agree
16d	I think my top choice has a good overall image.	Strongly disagree
		Disagree
		Neither agree nor disagree
		Agree
		Strongly agree
16e	I think my top choice requires a high level of skill.	Strongly disagree
		Disagree
		Neither agree nor disagree
		Agree
		Strongly agree
17a	I think primary care physicians have a good reputation in the community.	Strongly disagree
		Disagree
		Neither agree nor disagree
		Agree
		Strongly agree
17b	I think primary care physicians have a good reputation in the healthcare field.	Strongly disagree
		Disagree
		Neither agree nor disagree
		Agree
		Strongly agree
17c	I think primary care physicians are actively involved in serving the community.	Strongly disagree
		Disagree
		Neither agree nor disagree
		Agree
		Strongly agree
17d	I think primary care physicians have a good overall image.	Strongly disagree
		Disagree
		Neither agree nor disagree
		Agree
		Strongly agree
17e	I think primary care physicians require a high level of skill.	Strongly disagree
		Disagree
		Neither agree nor disagree
		Agree
		Strongly agree
18a	I think income is the most important factor when choosing a specialty.	Strongly disagree
		Disagree
		Neither agree nor disagree
		Agree
		Strongly agree
18b	I would not choose a specialty with a low income relative to other specialties.	Strongly disagree
		Disagree
		Neither agree nor disagree
		Agree
		Strongly agree
18c	Paying off student loans is my main priority after graduating medical school.	Strongly disagree
		Disagree
		Neither agree nor disagree
		Agree
		Strongly agree
18d	I want to make the most money working the least amount of hours.	Strongly disagree
		Disagree
		Neither agree nor disagree
		Agree
		Strongly agree
19	What is the minimum level of income you would be satisfied with as an attending?	$150-199k
		$200-249k
		$250-299k
		$300-349k
		$350-399k
		$400-449k
		$450k+
20a	A clinical experience made me want to choose a particular specialty.	Strongly disagree
		Disagree
		Neither agree nor disagree
		Agree
		Strongly agree
20b	A clinical experience made me want to AVOID a particular specialty.	Strongly disagree
		Disagree
		Neither agree nor disagree
		Agree
		Strongly agree
20c	An experience with a mentor made me want to choose a particular specialty.	Strongly disagree
		Disagree
		Neither agree nor disagree
		Agree
		Strongly agree
20d	An experience with a patient made me want to choose a particular specialty.	Strongly disagree
		Disagree
		Neither agree nor disagree
		Agree
		Strongly agree
20e	An experience with a patient population made me want to choose a particular specialty.	Strongly disagree
		Disagree
		Neither agree nor disagree
		Agree
		Strongly agree
20f	An experience with a patient population made me want to AVOID a particular specialty.	Strongly disagree
		Disagree
		Neither agree nor disagree
		Agree
		Strongly agree
21a	I am interested in art.	Strongly disagree
		Disagree
		Neither agree nor disagree
		Agree
		Strongly agree
21b	I am interested in music.	Strongly disagree
		Disagree
		Neither agree nor disagree
		Agree
		Strongly agree
21c	I am interested in reading and writing.	Strongly disagree
		Disagree
		Neither agree nor disagree
		Agree
		Strongly agree
21d	I am interested in cooking or baking.	Strongly disagree
		Disagree
		Neither agree nor disagree
		Agree
		Strongly agree
21e	I am interested in puzzles.	Strongly disagree
		Disagree
		Neither agree nor disagree
		Agree
		Strongly agree
21f	I am interested in video games.	Strongly disagree
		Disagree
		Neither agree nor disagree
		Agree
		Strongly agree
21g	I am interested in exercise or playing sports.	Strongly disagree
		Disagree
		Neither agree nor disagree
		Agree
		Strongly agree
